# Establishment of diagnostic reference levels in cardiac computed tomography

**DOI:** 10.1002/acm2.12711

**Published:** 2019-08-30

**Authors:** Mohammad Rawashdeh, Charbel Saade, Maha Zaitoun, Mostafa Abdelrahman, Patrick Brennan, Haytham Alewaidat, Mark F. McEntee

**Affiliations:** ^1^ Faculty of Applied Medical Sciences Jordan University of Science and Technology Irbid Jordan; ^2^ Department of Diagnostic Radiology American University of Beirut Medical Center Beirut Lebanon; ^3^ Faculty of Health Sciences Medical Image Optimization and Perception Group (MIOPeG), and the Brain and Mind Centre The University of Sydney Sydney NSW Australia; ^4^ Discipline of Diagnostic Radiography UG 12 Aras Watson Brookfield Health Sciences University College Cork College Road Cork Ireland

**Keywords:** CCT, CTDI_vol_, DLP, DRL, Jordan

## Abstract

The aim of this study was to determine diagnostic reference levels (DRLs) for cardiac computed tomography (CCT) in Jordan. Volume computed tomography dose index (CTDI_vol_) and dose–length product (DLP) were collected from 228 CCTs performed at seven Jordanian hospitals specialized in cardiac CT. DRLs for cardiac CT were defined at the 75th percentile of CTDI_vol_ and DLP. CTDI_vol_ and DLP were collected from 30 successive cardiac CT in each center except for one center (18 scans). The 75th percentile of the CTDI_vol_ and the DLP of the centers calculated from mixed retrospective and prospective gated modes were 47.74 milligray (mGy) and 1035 mGy/cm, respectively. This study demonstrated wide dose variations among the surveyed hospitals for cardiac CT scans; there was a 5.1‐fold difference between the highest and lowest median DLP with a range of 223.2–1146.7 mGy/cm. Differences were associated with variations in the mAs and kVp. This study confirmed large variability in CTDI_vol_ and DLP for cardiac CT scans; variation was associated with acquisition protocols and highlights the need for dose optimization. DRLs are proposed for CCT; there remains substantial potential for optimization of cardiac CT examinations for adults in Jordan.

## 1 INTRODUCTION

Radiation doses differ between hospitals for standard‐sized patients undergoing the same examination.^1^ These dose variations are “primarily attributable to local choices regarding technical parameters, rather than patient, institution, or machine characteristics.” Computed tomography (CT) has a large range of scanning options that lead to a large variation in patient radiation doses. Several factors affect radiation dose to patients undergoing cardiac computed tomography (CCT) procedures.^2^ Concerns have been raised about the radiation exposure of CCT. Recently, prospective gated mode (PGM) was developed to reduce radiation dose. PGM, also known as “step‐and‐shoot” or “sequential scan mode,”^3^ has been developed as an alternative mode to standard helical (spiral) scanning with retrospective electrocardiographic gating with the aim of decreasing radiation dose during CCT to patients with stable heart rates. Previous work by other authors has identified key contributors to variations in dose as radiographic settings, equipment factors, the level of quality assurance in place, radiographer training, radiographer experience, as well as patient body habitus.^4^, ^5^ Dose optimization requires identification of which factors are the greatest contributor to variations in dose. Once the contribution of factors is established, operators can consider corrective action in a cost‐effective manner.^4^

In order to achieve optimization, guidance on appropriate levels of patient exposure is required. Basic safety standards have been provided by the International Commission on Radiological Protection (ICRP), World Health Organization (WHO), and International Atomic Energy Agency (IAEA) in order to optimize the protection of patients during all radiological procedures, including CT.^6^ These include a recommendation for DRLs^7^ or guidance levels to be put in place, monitored, and used to improved radiological procedures.^8^ DRLs enhance patient protection and allow comparisons of the radiation dose of different CT systems, and procedures. DRLs offer a framework where dose levels from different hospitals can be compared and when DRLs are exceeded by a department, corrective strategies can be taken.

DRLs are not dose limits, but rather guidelines. Where they are regularly exceeded, corrective action should be taken. DRLs have been effective in reducing radiation dose, with radiation levels being reduced by 50% in the UK since their adoption.^9^, ^10^ As dose variation for the same examination can reach up to 23‐fold between centers for non‐CT X‐ray examinations^2^ and 13‐fold for CT examinations.^11^ Strategies such as DRLs are required to reduce dose variation between centers. The method of establishing DRLs starts with a determination of radiation dose levels delivered for specific examinations in several hospitals in an individual country (or state). These data are then used to compute examination‐specific DRLs for that country, state or region, usually in terms of the 75th percentile of the dose distribution. Due to the differing ethnicities, procedures, and equipment across different countries, it is advisable that each country determine their own DRLs. While the requirement for DRLs have been described in legislative documents in Jordan and internationally, and their implementation for general examinations is seen in Europe and the US, national DRL values do not exist for cardiac CT examinations in Jordan. Cardiac CT examinations are among the highest dose examinations, with patients undergoing chest CT having doses ranging from 4 to 18 mSv but those undergoing cardiac CT receiving 5–32 for a coronary CT angiogram, and 12–18 mSv for a 64‐slice coronary CT Angiography (CTA) with tube modulation. However, with good technique, a prospectively triggered coronary CTA patient can receive 2–4 mSv.^12^ The dose for cardiac CT in Jordan is currently not known, so we cannot compare our performance internally or internationally. The current work aims to address this gap.

## 2 MATERIALS AND METHODS

In 2018, a retrospective survey was performed across seven Jordanian hospitals that routinely perform CCT. Data from 228 cardiac CT scans were collected over a 3‐month period from May to August. These cardiac CT scans were performed on a wide range of scanners with different number of slices from three manufacturers (GE, Siemens, and Philips). These are representative of CT scanners being used across Jordan. According to the Jordanian regulations, CT scanners undergo a quality control program that includes daily, monthly, quarterly, and annual checking of the assessment of the radiation dose.^13^

Dose in CTDI_vol_ and DLP was provided in 30 successive cardiac CT scans in each center except for one, which provided 18 scans. There survey respondents used two scanning modes, prospective electrocardiographic (ECG) gating modes (PGM) where the scanner monitors the patient's ECG and retrospective ECG‐gating modes (RGM) where the patient is scanned continuously, while only certain portions of the scan are used to reconstruct the image. Participants were all adults between 17 to 75 yr and all genders were included. In line with a methodology previously published,^9^, ^14^ protocol and scan sequence details were grouped in the survey (Table 1). A clinical coordinator with CT experience was appointed in each center to administer and receive the questionnaires. The survey focused on CTA performed for the assessment of coronary artery disease. CTA included one single scan per examination. Calcium scoring, evaluation bypass graft patency and preparation of trans catheter aortic valve implantation were excluded.

**Table 1:** Information about the scanners in each hospital.

Hospital	Manufacturer	Year of manufacture	CT model type	Year of installation	Gantry rotation	Capacity (slice No.)
A	Philips	2008	Brilliance ICT scan 256	2011	360	256
B	Siemens	2014	Somatom force	2016	360	384
C	Philips	2010	Philips multislices 64p/s	2010	360	65
D	Siemens	2010	Multislices 128	2011	360	128
E	Siemens	2006	Somatom sensation 64	2006	360	64
F	GE	2011	Optima 99 multislice	2012	360	180
G	Siemens	2008	Somatom sensation 64 dual Source	2008	360	128

To standardize weight for the sampled population and in line with international recommendations, the survey included only those patients who weighed between 60 and 80 kg.^9^

### 2.1 Radiation dose data

On modern CT scanners CTDI_vol_ and DLP are provided for every sequence and examination. However, on older equipment it was necessary to calculate the dose using exposure and procedural data. The patient dose data, CTDI_vol_ and DLP values, were extracted from the picture archiving and communication system (PACS). A summary of the two relevant dose parameters is given below along with methods for calculating these factors. CTDI_vol_ is defined as weighted CTDI (CTDI_w_) divided by CT pitch and provide an estimation for average phantom dose for a complete spiral CT scan.^15^

### 2.2 Weighted CT Dose Index — CTDI_w_

This describes the radiation dose delivered per unit cranio–caudal (z) axis thickness. Significant previous work has provided methodologies so that the baseline dose value CTDI_w_ can be calculated for specific examinations and from this baseline value other important dose metrics can be calculated^4^, ^7^, ^16^, ^17^, ^18^, ^19^, ^20^, ^21^, ^22^ and these are explained below. Using typical CTDI_w_ values calculated from a dosimetry phantom for each CT scanner model, with specific exposure factors, the IMPACT group^14^ has provided a calculator from which CTDI_w_ can be calculated for any sequence, for any model using a range of exposure factors. This calculation is shown in Formula 1.

(1) $${\text{CTDI}}_{w}\left(\text{mGy}\right)={\left(n\;{\text{CTDI}}_{w}\right)}_{\text{scanner,phantom,kV,N}\times T}\times {\left(F_{N\times T}\right)}_{\text{scanner}}\times C$$

where (n CTDI_w_)_scanner, phantom, kV, NxT_ is a coefficient based on the normalized dose [CTDI_w_ for a specific scanner at a particular kV, slice number (N), and thickness (T)]; (F_NxT_)_scanner_ are dose coefficients for all other slice number and thicknesses; C is the mAs or effective mAs if pitch correction was included

### 2.3 Volumetric CT dose index ‐ CTDI_vol_

Since CTDI_w_ does not consider whether axial slices are contiguous, noncontiguous, or overlapping, a “pitch” correction must be added which provides a more representative volume CTDI or CTDI_vol_ (Formula 2).

(2) $${\text{CTDI}}_{\text{vol}}\left(\text{mGy}\right)=\frac{{\text{CTDI}}_{w}}{\text{Pitch}\;\text{factor}}$$

where pitch factor is the distance the table moves in the z axis divided by the slice number and each slice thickness.

CTDI_w_ is calculated from Formula 1.

### 2.4 Dose Length Product (DLP)

The dose measurements above do not consider the total length of the patient who has been irradiated during each examination sequence. This is calculated using Formula 3.

(3) $$\text{DLP}\left(\text{mGy}\cdot \text{cm}\right)={\text{CTDI}}_{\text{vol}}\times \text{Scan}\;\text{length}$$

where CTDI_vol_ is calculated from Formula 2.

### 2.5 Data analysis

The minimum, maximum, and the first, second, and third quartiles were calculated for CTDI_vol_, and DLP using Statistical Package for the Social Sciences (SPSS) software (SPSS for Windows, version 22.0, SPSS Inc.). Stepwise regressions were performed to find which exposure factors were associated with high dose and the level of contribution of each factor. The following factors were included: kVp, mAs, pitch, slice thickness, and number of slices. In addition, international DRLs were compared to the current national DRL level established in the current work.

## 3 RESULTS

Seven hospitals (four public, one university, and two private) participated in the study. Recordings from a total of 228 CCT examinations were collected, of which 60 CCT were performed using PGM and 168 using RGM. Sixty‐one percent of patients were males and 39% were females and the mean weight for both of them was 70.1 kg (Min = 60; Max = 80, SD = 8.93). Data were collected from seven scanners, of which two and five centers acquired image data in 256 and 128 slices. This represents approximately 31% of cardiac CT units in Jordan and exceeds the sample sizes used previously to establish reference levels in other countries.^5^, ^7^ A sample size calculation indicated a difference <5% in dose would be detected at 0.8 power. Patient and equipment characteristics and cardiac CT protocols are shown in Table 2. A summary of DRLs per scan mode (PGM, RGM, and Mixed Modes) and per center is shown Table 3.

**Table 2:** Patients and equipment's characteristics and cardiac computed tomography (CT) protocols (n = 228).

Characteristics	n (%) or mean (IQR)
Gender	
Male	139 (61.0%)
Female	89 (39.0%)
Age (years)	50.72 (41–61)
Weight (kg)	70.1 (66.2–80.0)
Body mass index (kg/m^2^)	30 (28–32)
Scan mode	
PGM	60 (26.3%)
RGM	168 (73.7%)
CT model (slice)	
Philips	59 (25.9%)
Siemens	138 (60.5%)
GE Optima 99	31 (13.6%)

**Table 3:** Diagnostic reference levels (DRLs) for mixed, prospective gating mode (PGM), and respective gating modes (RGM) cardiac computed tomography (CT) scans in Jordan.

Scan type	Mixed modes	PGM (n = 60)	RGM (n = 168)
CTDI_vol_			
Minimum	2.00	2.00	6.60
25th	13.71	4.50	21.00
Median	31.93	7.84	40.42
75th	47.74	33.37	64.54
Maximum	86.64	33.80	86.64
DLP			
Minimum	27.60	27.60	216.00
25th	329.58	151.80	431.97
Median	727.00	626.60	888.30
75th	1035.00	692.95	1141.50
Maximum	2865.00	740.00	2865.00

The mean CTDI_vol_ and DLP per CCT examination were calculated for each site and used to compare doses across CCT centers (Table 4). Wide variations were found between hospitals surveyed, with 1‐ to 5.1‐fold differences in mean CTDI_vol _and DLP reported for the examinations surveyed.

**Table 4:** Diagnostic reference levels (DRLs) for cardiac computed tomography (CT) scans performed with Mixed Modes per center.

Center	1 (n = 30)	2 (n = 45)	3 (n = 29)	4 (n = 45)	5 (n = 18)	6 (n = 31)	7 (n = 30)
CTDI_vol_							
Minimum	7.84	24.06	21.0	4.50	13.55	2.63	2.0
25th	10.78	38.85	31.93	4.50	13.55	64.54	5.80
Median	12.69	43.89	64.54	9.90	18.37	64.54	19.59
75th	19.15	47.87	79.43	33.80	22.30	64.54	21.70
Maximum	44.73	79.43	86.64	33.80	22.30	64.56	21.9
DLP							
Minimum	146.90	385.00	583.9	151.80	329.58	35.50	27.60
25th	189.10	807.40	831.20	618.10	329.58	1126.20	140.00
Median	223.20	888.30	1035.00	653.60	431.97	1146.70	251.05
75th	583.90	934.60	1435.00	726.50	1267.07	1236.80	293.00
Maximum	933.40	1441.90	1549.00	740.00	1267.07	1356.10	2865.00

Comparisons of the findings with DRLs published from other countries are shown in Table 5.^18‐24^

**Table 5:** Cardiac computed tomography (CT) diagnostic reference levels (DRLs) in Jordan compared with other international cardiac CT DRLs.

	Iran^18^	France^19^	Italy^23^	Netherlands^20^	Japan^21^	Switzerland^24^	KSA^22^	Jordan (current study)
CTDI_vol_								
Mixed	66.5	–	61	–	–	50	–	47.74
PGM	–	26	–	–	–	–	29	33.37
RGM	–	44	–	–	–	–	43	64.54
DLP								
Mixed	1073	–	1208	671	1510	1000	–	1035.0
PGM	–	370	–	–	–	–	343	692.95
RGM	–	870	–	–	–	–	808	1141.50

Multiple regression analysis for the Mixed Modes suggested that mAs, kVp, and number of slices were more accurately predictive of CTDI_vol_ than any individual variable alone, with R^2^ of 0.530 (*P* ≤ 0.001). The results of the regression also showed that combination of mAs, kVp, and number of slices could significantly predicate DLP. All factors had a significant positive predictor value, with a comparatively low R^2^ of 0.364 (*P* ≤ 0.001). The equations were

$${\text{CTDI}}_{\text{vol}}=-72.00+0.03\;A+0.800\;B-0.013\;C$$

where A is mAs, B is kVp, and C is the number of slices.

DLP=-1352.19+0.549A+16.794B-0.233C

where A is mAs, B is kVp, and C is the number of slices.

In the PGM mode, the results from the multiple linear regressions demonstrated that mAs was more accurately predictive of CTDI_vol_ than any other variable, with R^2^ of 0.646 (*P* ≤ 0.001), while for the case of the DLP, kVp, number of slices, and mAs could significantly predicate DLP, with R^2^ of 0.820 (*P* ≤ 0.001), which is significantly higher (Table 6).

$${\text{CTDI}}_{\text{vol}}=-8.238+0.070\;A$$

where A is mAs

DLP=-1475.37+17.685A-1.049B+0.750C

where A is kVp, B is the number of slices, and C is mAs

**Table 6:** Predictive of CTDI_vol_, and dose–length product (DLP) from exposure factors using stepwise regression factors for Mixed Modes, prospective gated mode (PGM), and retrospective ECG‐gating modes (RGM).

	R^2^	*P*‐value
Mixed modes		
CTDI_vol_		
mAs	0.414	≤0.001
kVp	0.496	≤0.001
Number of slices	0.530	≤0.001
DLP		
mAs	0.243	≤0.001
kVp	0.335	≤0.001
Number of slices	0.364	≤0.001
PGM		
CTDI_vol_		
mAs	0.646	≤0.001
DLP		
kVp	0.585	≤0.001
Number of slices	0.711	≤0.001
mAs	0.820	≤0.001
RGM		
CTDI_vol_		
kVp	0.342	≤0.001
Pitch	0.506	≤0.001
mAs	0.600	≤0.001
Number of slices	0.625	≤0.001
Slices thickness	0.635	≤0.001
DLP		
mAs	0.167	≤0.001
kVp	0.232	≤0.001
Number of slices	0.268	≤0.001

For the RGM mode, the results from the multiple linear regressions demonstrated that kVp, pitch, mAs, number of slices, and slice thickness were the predictive factors of CTDI_vol_, with R^2^ of 0.635 (*P* ≤ 0.001), while mAs, kVp, and number of slices were the predictive factors of DLP with R^2^ of 0.268 (*P* ≤ 0.001), which is relatively lower as demonstrated in the following two formulas.

$${\text{CTDI}}_{\text{vol}}=-57.091+0.908A-30.433B+0.023C-0.011D-4.307E$$

where A is kVp, B is pitch, C is mAs, D is the number of slices, and E is the slice thickness.

DLP=-1244.8+0.417A+17.03B-0.228C

where A is mAs, B is kVp, and C is the number of slices.

## 4 DISCUSSION

DRLs were first proposed by the International Commission on Radiological Protection in 1991. They are defined as “dose levels in radio diagnostic practices for typical examinations for patient groups or standard phantoms for broadly defined groups of equipment.”^9^, ^25^, ^26^ Patient radiation doses that exceed established DRLs should be investigated to identify potential reasons for higher dose and to allow better management of the radiation dose of similar procedures in the future.^21^, ^27^ With the significant amount of studies that have been conducted to establish the DRL levels in other countries and in the different CT scans,^4^, ^7^, ^16^, ^17^, ^18^, ^19^, ^20^, ^21^, ^22^ this is the first study to establish DRLs in CCT in Jordan.

Research to date has demonstrated a maximum potential dose reduction in CT scan between 60% and 80%.^28^, ^29^, ^30^, ^31^ However, it is important to appropriately balance the need to achieve low radiation dose with the likelihood of creating useful diagnostic CT images. Low radiation exposure for a certain patient during CCT scan may result high image noise; however, it needs to be acknowledged that while high radiation exposure may increase image quality and reduce image noise, this does not automatically mean additional diagnostic information.^32^ The current work highlighting wide dose variations for cardiac CT scans were shown across hospitals. With a range of 223.2–1146.7 mGy cm, the highest mean DLP was 5.1 times higher than the median value. These differences were primarily attributable to local choices regarding technical parameters, rather than patient, institution, or machine characteristics. Changes in CTDI_vol_ were associated with variations in the mAs, kVp pitch, and slice thickness. Multiple regression analysis predicted that low DLP was most dependent on mAs, kVp, mode of scan, and number of slices.

The current study reports that PGM allowed a significant dose reduction with CTDI_vol_ of 60.4% compared with the RGM mode. These wide dose variations between modes emphasize the need to analyze the CTDI_vol_ and DLP individually and, therefore, establish DRL for each mode.^22^ The current work did not compare the diagnostic performance of PGM with RGM. However, the reduction in radiation dose with PGM scanning was larger than the effect of other radiation dose‐reduction techniques. The 75th DLP in scanned average‐sized patients was only 692.95 mGy cm. Among those centers using PGM, the lowest median DLP was 223.2 mGy cm in Centre A, this dose was in contrast with 75th DLP of RGM 1146.60 mGy cm. This finding supports the use of PGM as an effective tool in comprehensive radiation dose‐reduction technique. The current work shows that a reduction of 26.3% in the DLP in CT scanning within participating centers is achievable with PGM, this is a larger decrease in dose than reported in other works.^2^, ^19^, ^22^

Compared with nationwide surveys from other countries, Jordanian CCT centers generally appear to employ higher doses than those countries previously surveyed; therefore, there is a large potential for optimization of CCT examinations for adults in Jordan. Variation in radiation dose shown in the current work highlights the need for the adoption of DRLs that radiologists or radiographers need to optimize their CCT protocols and that interest in dose optimization must be improved. The work also demonstrates the need for periodic reassessment of DRLs at short‐time intervals. Clinical audits should also identify further causal agents, eliminate unjustified examinations, and optimize procedures.

Further work should investigate size‐specific dose estimate (SSDE). SSDE accounts for patient parameters establishment of DRLs and removes the requirement to limit the average of the sample to between 60 and 80 kg. Additional studies should to be conducted on the SSDE application to DLP so that scan length can be considered for in the equation of patient dose.

There are several noteworthy limitations to our study. First, as our analysis was retrospective, we could not obtain information on several parameters that possibly influence the radiation dose such as beam collimation, rotation time, and patients' diameter. Second, we did not assess the CT scan indications; hence, the parameters of these CT scans may not represent the routine protocols of the respective institutions. Third, since our analysis was conducted on only seven institutions, bias could have been introduced, even though the CT scans we obtained were from various centers geographically dispersed throughout Jordan.

This study demonstrates large variability in CTDI_vol_ and DLP during CCT examinations in Jordan and highlights the need for doses to be reduced. PGM is clearly an effective dose‐reduction technique for cardiac CT examinations and the use of this mode should be encouraged. Local DRL results should be communicated back to each CT center to encourage optimization of scan parameters and develop more proactive comparisons with national DRL and other CT centers.

## CONFLICT OF INTEREST

There is no conflict of interest declared in this article.
